# Efficacy of Praziquantel against *Schistosoma haematobium* in Dulshatalo village, western Ethiopia

**DOI:** 10.1186/1756-0500-6-392

**Published:** 2013-09-30

**Authors:** Asaye Mekonnen, Mengistu Legesse, Mulugeta Belay, Konjit Tadesse, Workineh Torben, Zelalem Teklemariam, Berhanu Erko

**Affiliations:** 1 School of Medical Laboratory Sciences, Medical faculty, Addis Ababa University, P.O. Box 180056, Addis Ababa, Ethiopia; 2 Aklilu Lemma Institute of Pathobiology, Addis Ababa University, P.O. Box 1176, Addis Ababa, Ethiopia; 3 Department of Medical Laboratory Sciences, College of Medical Sciences, Haramaya University, Harar, Ethiopia

**Keywords:** Efficacy, Praziquantel, S. haematobium

## Abstract

**Background:**

Praziquantel (PZQ) is the drug of choice for treatment of all human schistosomes. It is used in population based targeted or mass deworming strategies in several countries. The effect of PZQ on S. hematobium has not been studied in Ethiopia. The objective of this study was to determine the efficacy of PZQ against *S. haematobium* in Dulshatalo village, western Ethiopia.

**Methods:**

A prospective study was conducted from October to December, 2007. Urine samples from 341 residents were collected and screened for haematuria and proteinuria using urinalysis dipstick. *S. haematobium* eggs were detected and quantified using filtration techniques. The participants who were positive for haematuria were treated with a standard dose of PZQ (40 mg/kg). Data on pre and 24 hours post treatment symptoms were collected via questionnaire. Urine samples were also collected 7 weeks after treatment and examined to assess the cure and the egg reduction rates.

**Results:**

The prevalence of *S. haematobium* among the study participants was 57.8% (197/341). Haematuria was detected in 234 (68.6%) of the study participants. For PZQ efficacy asessment, 152 of the treated participants were considered. The presence of *S. haemetaobium* eggs showed statistically significant association (p < 0.05) with haematuria and proteinuria. Seven weeks post treatment, the extent of haematuria and proteinuria decreased from 100% to 40.8% and 94.07% to 48.7%, respectively. The cure and the parasitological egg reduction rates seven weeks post treatment were 86% and 85%, respectively. Post treatment symptoms revealed a wide range of side effects including straining, abdominal pain, nausea and headache.

**Conclusions:**

There were marked cure and egg reduction rates, together with mild and short lived side effects of PZQ for treatment of *S. haematobium,* in this study.

## Background

Schistosomiasis is the second most prevalent tropical disease next to malaria in the world. It affects about 200 million people worldwide with more than 650 million people living in endemic areas [1-3]. A recent survey in sub-Sahara Africa indicates that 70 million individuals out of 682 million have experienced haematuria with 32 million cases of dysuria associated with *S. haematobium* infection. Furthermore, it was estimated that 18 million people suffer from *S. haematobium*-related bladder wall pathology and 10 million from hydronephrosis [3]. In Ethiopia, *Schistosoma haematobium* is found in the middle, lower Awash [4], lower Wabe Shebelle valleys [5] and Kumruk on the Ethio-Sudanese border [6].

Schistosomiasis can be controlled via chemotherapy, snail control, improved sanitation, and health education. Currently, a population based target/mass chemotherapy with Praziquantel (PZQ) is a major controlling strategy in several countries [7-9]. PZQ is the drug of choice for schistosomiasis control due to its effect on the adult stages of the five species of human schistosomes, minimal side effects [10-12], easy administration and tolerability [13-15]. On the other hand, it lacks efficacy against the immature stages of schistosomes which is thought to be a risk for resistance development [16].

The efficacy of PZQ on schsitosomes has been studied in many countries. Most of the studies made are on its efficacy in the treatment of S*. mansoni* and have reported different cure and egg excretion reduction rates [17-26]. In the treatment of *S. haematobium*, low cure rates are reported from Ghana [27] and Senegal [28], while higher cure and egg reduction rates are obtained from Côte d'Ivoire [29], Cameroon [30], Zimbabwe [31] and South Africa [32]. The explanations forwarded for this variation in efficacy includes the issue of drug resistance [19], and presence of pre-patent infections existing at the time of treatment [33] in high transmission areas and the inadequacy of single dose of 40 mg/kg under conditions of extremely high intensity of *S. haematobium* infection [33].

Side effects due to PZQ usually occur in a relatively larger population of patients (30-60%) but they are mild and transient and disappear within 24 hrs [14,25]. Dizziness, bloody or mucoid diarrhoea and abdominal pain are most frequently encountered following the treatment of infections with *S. mansoni* in studies conducted in Cotedivore, Uganda and North-East Ethiopia [22,23,25,29]. Vomiting, weakness and head ache are reported from North-East Ethiopia [25]. On the other hand, uncommon side effects such as joint pains, joint swellings, and myalgia and peri-tibial/ankle oedema are observed following treatment of *S. haematobium* in Afar Region, eastern Ethiopia [34].

Monitoring of PZQ efficacy in different areas, where the drug is widely used is important. This is because of the absence of alternative drug for the effective treatment of schistosomiais, the presence of genetic differences in the schistosome strains and diverse reports in efficacy of the drug world-wide. In Ethiopia, PZQ is used for the treatment of cestodes and trematodes at different regimen. It is also used in mass treatment of school children and the community. However, the efficacy of PZQ against Ethiopian strain at different foci is not well investigated. Therefore, the efficacy of PZQ was researched in Dulshatalo village, one of the urinary schistosomiasis endemic areas in Ethiopia.

## Methods

### Study design, area and period

A prospective study was conducted from October to December, 2007 in Dulshatalo, a village located about 878 Km west of Addis Ababa and near Ethio-Sudanese border. Dulshatalo is one of the urinary schistosomiasis endemic areas in Ethiopia. It is 800 m above sea level and has population of 801 people [35]. Most of the residents lived in the area since birth and are involved in traditional gold mining. There are two water bodies in the village: a pond and a stream. The people are mostly dependent on these sources of water for bathing, laundry and traditional gold mining activities.

### Sample size and sampling techniques

Sample size was not determined. All of the residents were summoned for urine examination. Three hundred forty one of them presented themselves for screening.

### Data collection and measurement

#### Urine collection and analysis

Initially the socio-demographic and clinical data were collected from study participants. Then, they were given a wide, clean, labeled container to provide terminal urine between 10.00 a.m. and 2.00 p.m. Since haematuria is the best indicator of urinary schistosomiasis in an endemic area [8], the participants were tested on the spot using reagent strips (URS-11, Teco diagnostics, USA). Preserved in 0.2 ml of 37% formalin, the urine samples transported to Aklilu Lemma Institute of Pathobiology (ALIPB) laboratory and then examined, using filtration technique for *S. haematobium* eggs. The intensity of infection was assessed according to guideline developed by Montresor *et al*. [8]. To assess the cure and the egg reduction rates [8], urine samples were collected from the study participants whose urine samples were positive for ova of *S. haematobium* before treatment, who were treated with PZQ and who were able to report for re-examination, seven weeks post-treatment.

#### Treatment

The study participants were treated with single dose of PZQ (Biltricide®, Bayer AG, Germany), 40 mg/kg body weight which is a standard treatment dose in Ethiopia.

#### Clinical diagnosis

compliance about symptoms and side effects of the drug were collected pre and 24 hrs post treatment, using questionnaires.

### Data analysis

Data from the questionnaire and parasitological study were double entered into excel. Statistical analysis was done using STATA Version 8.1(Stata Corporation, College station, Texas, USA). Simple frequency and proportions were used to describe the data. Chi-square (for categorical variables) and correlation (for continuous variables) were used to evaluate associations. Values were considered statistically significant when p-value < 0.05 at 95% confidence interval.

The cure rate was the proportion of *S. haematobium* infected patients who had no egg excretion six weeks after treatment.

The egg reduction rate was calculated as the reduction in the intensity of infection assessed indirectly, using egg count via the following formula (8):

$$\begin{array}{l} \%\;\text{egg}/10\mathrm{ml}\;\text{reduction}=\left(1-\text{Geometric}\;\text{mean}\;\text{of}\;\text{eggs}/10\mathrm{ml}\right)\\ \left(\;\text{urine}\;\text{after}\;\text{treatment}\right)x\;100\;\text{Geometric}\;\text{mean}\\ \;\text{of}\;\text{eggs}/10\mathrm{ml}\;\text{urine}\;\text{before}\;\text{treatment} \end{array}$$

### Ethical consideration

Ethical clearance was obtained from Ethical Clearance Committee of ALIPB, Addis Ababa University. The study participants involved in the study after they had given their informed consent. For the participants younger than 18 years, consent was obtained from their parents or guardians.

## Results

### Study participants

A total of 341 participants (72.14% male) were involved at the initial screening of urine. Their age ranged from 2 to 60 years, with a median and a mean of 15 and 19 years, respectively. For PZQ efficacy study, 152 participants were enrolled, of whom 123 (80.9%) were male. Their age rangedfrom 2 to 60 years with a median and a mean of 13 and 16 years, respectively.

### Prevalence and intensity of *S. haematobium*

The overall prevalence of *S. haematobium* among the study participants was 57.8% (197/341), with an overall geometric egg count of 15.32 (Table 1). The presence of *S. haematobium* eggs was significantly associated with age (*x*^2^ = 20.12, p = 0.003), haematuria (*x*^2^ = 63.83, p < 0.001), and proteinuria (*x*^2^ = 32.94, p < 0.001) (Table 1), but not with sex (Table 1). In addition, the presence of *S. haematobium* eggs in urine was correlated with the degree of haematuria (r = 0.2108, p = 0.0001) and of proteinuria (r =0.2171, p = 0.0001).

**Table 1:** **Prevalence and Intensity of *S. haematobium *based on sex,age, haematuria and proteinuria in Dulshatalo village, western Ethiopia**

**Variable**		**Total examined**	**Intensity of infection**			***x***^**2**^	**p-value**
*Sex*			Light(<50 eggs/10 ml)	Heavy(≥50 eggs/10 ml)	Total		
	Male	246	108	38	146		
	Female	95	41	10	51		
	Total	341	149	48	197	0.85	0.36
	Geometric mean				15.32		
Age							
	< 5	14	4	1	5		
	5-14	150	67	37	104		
	15-24	90	43	2	45		
	25-34	43	17	3	20		
	35-44	23	10	1	11		
	≥ 45	21	8	4	12		
	Total	341	149	48	197	20.12	0.003
Haematuria							
	Yes	234	122	47	169		
	No	107	27	1	28		
	Total	341	149	48	197	7.66	0.006
Proteinuria							
	Yes	259	125	47	172		
	No	82	24	1	25		
	Total	341	149	48	197	6.44	0.01

### *S. haematobium* infections before and after seven weeks of treatment with PZQ

Of the 152 participants treated with PZQ, 132 had no ova of *S. haematobium* in their urine samples after 7 weeks of treatment, which was an overall cure rate of 86%.

Parasitological egg count reduction of 85% after 7 weeks of treatment was observed. The number of eggs for every 10 ml urine ranged from 1 to 1488 with overall geometric mean of 19.95 eggs per 10 ml before treatment and this was markedly reduced to range of 1 to 50, with a geometric mean of 3.06 eggs per 10 ml after treatment (Table 2).

**Table 2:** Prevalence and intensity of S. haematobium before and seven weeks after administration of PZQ based on sex, age and geometric mean egg count in Dulshatalo village, western Ethiopia

**Variable**		**Intensity of infection before treatment**			**Intensity of infection**		
					**After treatment**		
		**Light (<50 Eggs/10 ml)**	**Heavy (≥50 eggs/10 ml)**	**Total**	**Light (<50 eggs/10 ml)**	**Heavy (≥50 eggs/10 ml)**	**Total**
Sex	Male	87	36	123	13	1	14
	Female	21	8	29	6	0	6
	Total	108	44	152	19	1	20
Age (Years)							
	< 5	3	1	4	0	0	0
	5-14	52	33	85	10	1	11
	15-24	35	3	38	5	0	5
	25-34	8	2	10	2	0	2
	35-44	8	1	9	1	0	1
	≥ 45	1	5	6	1	0	1
	Total	108	44	152	19	1	20
GM(eggs/10 ml)		8.20	176.67	19.95	2.64	50	3.06

### Haematuria and proteinuria before and after seven weeks of treatment with PZQ

All of the study participants (N = 152) had haematuria whereas 143 (94.07%) had proteinuria when examined using reagent strip tests before treatment. The prevalence of haematuria and proteinuria after 7 weeks of treatment declined to 40.8% and 48.7%, respectively (Table 3).

**Table 3:** Haematuria and proteinuria before and seven weeks after the administration of PZQ, Dulshatalo village, western Ethiopia

**Variable**		**Before treatment**		**After treatment**	
		**Haematuria (pos for Ova)**	**Proteinuria (Pos for Ova)**	**Haematuria (Pos for Ova)**	**Proteinuria (Pos for Ova)**
Sex	Male	123(123)	115(115)	53(8)	57(7)
	Female	29(29)	28(28)	9(4)	17(4)
	Total	152(152)	143(143)	62(12)	74(11)
Age (Years)					
	< 5	4(4)	4(4)	2(0)	2(0)
	5-14	85(85)	82(82)	35(7)	40(7)
	15-24	38(38)	35(35)	19(4)	22(3)
	25-34	10(10)	10(10)	2(0)	2(0)
	35-44	9(9)	7(7)	2(0)	6(0)
	≥ 45	6(6)	5(5)	2(1)	2(1)
	Total	152(152)	143(143)	62(12)	74(11)

### Pre and post treatment symptoms

Pre-treatment symptoms profiles were recorded from all of the study participants but only 65 out of 197 treated, reported at 24 hours post treatment and of them, fifty (76.92%) were male. All of the participants reported more than one symptom both before and 24 hrs post treatment.

Straining, diarrhoea, fatigue, drowsiness, fever and itching occurred with a higher frequency after treatment than before treatment, while headache and nausea which were common before treatment, declined. On the other hand, all the study participants reported haematuria before and 24 hours post treatment (Table 4).

**Table 4:** **Frequency of symptoms before and 24 hrs after treatment for *S. haematobium *with a single dose of PZQ, Dulshatalo village, western Ethiopia**

**Symptoms**	**Pre-treatment**		**24 hrs post treatment**	
	**Number**	**Percentage (%)**	**Number**	**Percentage (%)**
Haematuria	65	100	65	100
Straining	47	72.31	56	86.15
Abdominal pain	61	93.84	34	52.31
Vomitting	2	3.08	8	12.31
Nausea	54	83.07	9	13.85
Diarrhoea	0	0	11	16.92
Bloody stool	2	3.08	4	6.15
Fatigue	1	1.53	9	13.85
Headache	39	60	29	44.62
Dizziness	16	24.61	10	15.38
Drowsiness	0	0	8	12.31
Fever	0	0	19	29.23
Itching	0	0	26	40
Dysuria	0	0	23	35.38

## Discussion

All of the study participants belonged to the same ethnicity and religion and most have lived in the area since birth. Macrohaematuria was a common phenomenon in this community and it is indicative of high morbidity [8].

Our study revealed that the prevalence and the infection intensity according to WHO is categorized as high and indicates universal treatment is necessary to the community irrespective of age, sex, infection status, or other social characteristics [8].

In this study, a cure rate of 86.8% was found 7 weeks post- treatment, which is comparable to the findings of similar studies conducted in Cameroon [30], Zimbabwe [31], north-east Ethiopia [25]. The egg count reduction observed 7 weeks after treatment was 84.66%. This is lower than those observed in Cameroon [30], Zimbabwe [31], and north-east Ethiopia [25]. This might have been due to the presence of pre-patent infections [33] and lack of PZQ efficacy against the immature stages of schistosomes [16]. We haven’t determined the viability of excreted eggs and this is one limitation of our study. Dead eggs that may be present for months in the urine of treated patients infected with *S. haematobium *[10] will falsely decrease the egg reduction and cure rate results.

Higher cure rates were found in males than in females. Females in this community are responsible for gold mining,which exposes them to high water contact (for separating the gold out of the soil) which is infested with cercaria of schistosomes. This may lead to existence of pre-patent infections at the time of treatment. In addition, school age children had high prevalence and intensities of schistosome eggs and showed lower cure rates than adults. Since the children had an increased water contact due to different activities like swimming, pre-patent infections may exist at the time of treatment, which affects the cure and the egg reduction rates.

In response to treatment with single dose of praziquantel, haematuria fell from 100% to 40.8% and proteinuria from 94.07% to 48.7% seven weeks post- treatment. Most studies conducted have assessed effect of PZQ on haematuria and proteinuria over a period of months not in weeks as in the present study. A study in Kenya has found a decline of haematuria from 75% to17% and proteinuria from 73% to 27% 12 months after treatment [36]. A study from Ghana on the other hand has reported the reduction of haematuria by 77% in children less than 15 years of age and by 68% in adults at six month follow up [27].

Since all the residents of the village were Muslims and the data were collected in Ramadan (fasting month),most of them could not appear for interview on post-treatment symptoms and this is the second limitation of our study. But, the side effects reported in this study were more or less consistent with those side effects reported in other schistosome endemic areas [22,23,25,29]. Increased rates of straining, diarrhoea, fatigue, drowsiness, fever and itching were observed 24 hours after treatment. On the other hand, uncommon side effects such as joint pains, joint swellings, myalgia and peri-tibial/ankle oedema were not reported in this study as they were reported from Afar Region of Ethiopia in treatment of *S. haematobium* infected patients [34] and in treatment of *S. mansoni *[25] from north -east Ethiopia. Most of the post treatment symptoms manifested soon after drug therapy and the majority were mild and short-lived. On the other hand, the presence and degree of haematuria and proteinuria before treatment were correlated with the presence of *S.haeamtobium* eggs and count in urine, which is a similar finding with the study conducted in Kenya [36].

## Conclusions

Though PZQ was used for long time at Dulshatalo village for the treatment of schistosomiasis, the disease remains high. This study has indicated that in a highly endemic area with very high infection intensity, the cure and the egg count reduction rates at the recommended dose of 40 mg/kg were high. Mass chemotherapy together with the control of intermediate host snails and their habitats should be integrated into primary health care in the area to reduce the burden of the disease.

## Abbreviations

PZQ: Praziquantel.

## Competing interests

All authors declare that they have no conflict of interest associated with the publication of this manuscript.

## Authors’ contributions

AB participated in proposal write up, data collection, analysis, interpretation, drafting and critical review of the manuscript. All other authors participated in data collection, analysis, and interpretation and critical review of the manuscript. In addition ML, MB and BE participated in proposal write up. All of them read and approved the final manuscript.
